# An Exploration of Online Positive Feedback in Relation to Mental Health Nursing Practice

**DOI:** 10.1111/inm.70116

**Published:** 2025-08-10

**Authors:** Mark Pearson, Stefan Rennick‐Egglestone

**Affiliations:** ^1^ University of Nottingham Nottingham UK; ^2^ School of Health Sciences Institute of Mental Health, University of Nottingham Nottingham UK; ^3^ NIHR Nottingham Biomedical Research Centre Institute of Mental Health Nottingham UK

**Keywords:** mental health, nurse education, nursing

## Abstract

Web‐based databases of service user feedback have become an important resource for the task of understanding the quality of healthcare provision, both in the UK and internationally. This research explores positive feedback submitted to the Care Opinion website (https://www.careopinion.org.uk/), some of which can provide insights into effective mental health nurse practices. An iterative search was undertaken using the Care Opinion website on 7 March 2025. A set of tags which frequently identified effective mental health nursing practices was identified, and then 51 items of feedback were taken forward for full analysis. The data was then analysed in relation to the deductively selected domains of tone, form, and intent. These were identified in a typology produced by a previous study of 200 positive feedback items across multiple sources. In relation to tone, most pieces of feedback were entirely positive with a small number being mixed. The intent of the feedback was often to express gratitude but also functioned to emphasise change and contrast the helpfulness of certain staff or organisations against others which were experienced as less helpful. A typology of form was established, comprised of (1) Narratives of being there; (2) Narratives of listening and being heard; Narratives of therapeutic relationships; and Narratives of recovery. Positive feedback can provide a valuable insight into the experiences of service users. This research provides proof of concept evidence that knowledge can be gained about the impact of mental health nursing through the analysis of online feedback gathered through websites such as Care Opinion.

## 1 Introduction

In the UK and internationally, service user and carer feedback has become an integral element of understanding the quality and person‐centredness of healthcare provision for healthcare organisations and professionals (Donetto et al. 2019; Powell et al. 2019). Patient‐reported indicators of both outcomes and experiences have become increasingly prevalent as benchmarks of service quality and experience (de Bienassis et al. 2021), and the benefits of health services acknowledging and acting on service user feedback to improve patient care are widely accepted (Griffiths and Leaver 2018).

There has been a growth in online feedback and web‐based public facing feedback reporting platforms such as Care Opinion (https://www.careopinion.org.uk/). These web‐based spaces have transformed the way in which people are able to evaluate health services and publicly share their feedback on lived experiences (Baines et al. 2018).

Historically, the focus from organisations has been on service user feedback identifying concerns and potential shortcomings within healthcare (NIHR Dissemination Centre 2019). In recent years the failings of mental health services have been highlighted in the UK media, following several tragic high‐profile incidents, leading to the profession of mental health nursing becoming scrutinised in relation to cultures of abuse and restrictive practice within inpatient environments (Haslam 2024).

However, despite the proliferation of negative feedback, service users and carers often provide positive feedback (Viertiö et al. 2025) and increasingly there is a focus on understanding this positive feedback and how it can enable health service improvement (Lloyd et al. 2023, 2024). Therefore, this research examines positive online feedback directly relating to mental health nurses. The research aims to understand the nature of the positive feedback and identify the aspects of mental health nursing practice that are experienced as most meaningful by service users and carers.

## 2 Method

### 2.1 Approach

The research adopts a narrative methodology, considering each piece of feedback to be an experiential story of engaging with mental health nurses. Narratives as research objects provide insights into how people understand their social worlds (Bruner 1987), and thus provide an excellent insight into the ways in which mental health nurses have featured within these social worlds and the meanings associated with these events. As proposed by Andrews et al. (2008), narratives are sequential and meaningful, display transformation and change, and ‘re‐present’ human experiences, reconstituting them as well as expressing them.

Ethical approval was prospectively granted by the Faculty of Medicine and Health Sciences research ethics committee at the University of Nottingham, reference number: FMHS 291–0924.

### 2.2 Data Collection

A search was undertaken using the Care Opinion website on 7 March 2025. Care Opinion is an online service hosted by a not‐for‐profit organisation. It collects and publishes anonymous feedback on experiences of UK‐provided statutory health services and allows National Health Service (NHS) units to publicly respond to its contents, for example, by describing changes to service delivery that have been made. No permission is required for access to feedback or health service responses collected by Care Opinion, as all are published under the Creative Commons CC‐BY‐NC‐SA 4.0 licence, which allows for non‐commercial re‐use (https://creativecommons.org/licenses/by‐nc‐sa/4.0/deed.en).

Internet mediated research is associated with specific ethical issues, such as consent, anonymity, and the extent to which online posts are considered private (Phenwan et al. 2021; Whitehead 2007). However, the authors argue that the process through which Care Opinion gathers and displays data meets many of the criteria detailed in the guidance on internet mediated research written by The British Psychological Society (2021). The Care Opinion website gathers feedback which is clearly intended to be made public and requires all those who are planning to leave feedback to read an information page prior to writing feedback. The information page details what will happen to the feedback provided and how this information will be shared with healthcare organisations and professionals. Care Opinion is also transparent about its moderation policy which governs what is published, ensuring that all feedback items are authentic, public, legal, and safe for service users. Therefore, all direct quotes reported in the paper are written alongside links to the original piece of feedback on the Care Opinion website, and no names have been removed from the quotes.

Care Opinion allows the public to search for feedback by named tags, which are short pieces of text, composed of one or more words. When a piece of feedback is uploaded by an author, then both the author and a Care Opinion moderator can annotate it with tags. These frequently relate to health conditions, types of services accessed, or the emotional content of the feedback. Members of the public can then construct searches using one or more combinations of tags, along with other terms that they specify. In a study of feedback provided through Care Opinion and also the NHS Friends and Family Test, Lloyd et al. (2024) have demonstrated how searches can be constructed that are efficient at identifying large quantities of feedback containing positive content about health service provision. They called their searches “heuristics”.

In our own work, we began to develop searches by exploring feedback terms associated with mental health nursing. The results were then checked to clarify what ‘'tags’ were associated with feedback items found to contain positive content. Tags were then combined into a single search to create a heuristic with the greatest likelihood of turning up the feedback the research team were interested in for the purpose of this study.

This search generated 78 items of feedback. These 78 items were then individually read and screened to check for relevance to the topic. Following screening, 51 items of feedback remained and were taken forward for full analysis. Items were excluded due to either not being positive pieces of feedback or being pieces of feedback which, although positive, were predominately focused on other members of mental health services, such as occupational therapists or psychologists.

### 2.3 Data Analysis

All data was imported to NVivo and subjected to a three‐stage process of analysis based on three top‐level dimensions in a positive feedback typology produced by Lloyd et al. (2024): tone, form, and intent. These categories were applied deductively, but subcategories within these domains were inductively developed from the data, since Lloyd et al. (2024) acknowledge that their typology is incomplete. Firstly, the data was examined to determine if the tone of feedback was entirely (a) positive, or (b) mixed positive and negative. The intent was then determined by considering the purpose of the feedback from the perspective of the narrator who had produced it. For example, was the feedback designed to display gratitude or to highlight the benefits of services? The form of the data was then examined through a thematic narrative analysis with a focus on the semantics and content of each piece of feedback. This process of form analysis began with detailed immersion in the feedback narratives before beginning to develop the themes. Themes were then developed by focusing on the entire narrative within a single piece of feedback, and being attentive to the sequence and progression of the narrative. The process of analysis did not attempt to identify themes within each narrative but rather to identify the overall theme of each piece of narrative feedback. Once initial themes were developed, these were discussed and refined by the research team and developed into a typography.

### 2.4 Reflexivity

The lack of context for items of data derived from online platforms can pose a significant challenge to researchers (Pousti et al. 2021). The absence of a participant with whom to check the context presents the possibility of researchers assuming contexts or meanings which may be a departure from the original intentions of the author (Paulus et al. 2024). Throughout data analysis, the lead researcher was mindful of the need for positional/membership reflexivity (Whitaker and Atkinson 2021) due to their background as a mental health nurse. Engaging in reflexive discussions on the emerging findings helped to refine the process of data analysis and prevent assumptions by the research team. Moreover, by focusing the analysis on the three dimensions of tone, intent, and form, the researchers were able to examine the details of the feedback from different perspectives to limit the risk of the research team imposing assumptions on the data.

## 3 Findings

The below table presents the findings from the analysis of tone and intent.

**Table:** 

Dimension	Category	Category definition	Number of pieces of feedback
Tone	Positive only	Unambiguously positive feedback about either healthcare staff or service	43
	Mixed	A mixture of positive and negative feedback about healthcare staff or service	8
Intent	Expressing Gratitude	Expressions of gratitude related to the positive effect of individuals or teams	38
	Emphasising change	Highlighting the impact of individuals or teams within a person's life	6
	Contrasting helpfulness	Contrast the helpfulness of individuals or teams against less helpful alternative experiences	7

### 3.1 Tone

Most of the feedback was unambiguously positive and contained no negative comments; in fact, some pieces of feedback emphasised this specific point in stating that they had no negative feedback to offer.

My Community Psychiatric Nurse (CPN) comes once a week, every week; I often talking about difficult things to her and she is just so understanding and compassionate yet stays professional; always!

I couldn't wish for better care than what I receive, always going above and beyond to help and support me no matter what!

Thank you Community Mental Health Team (CMHT) Central” (https://www.careopinion.org.uk/1257450)

Some of the positive feedback was written as a direct communication to the person or organisation being commended within the feedback

I want to put forward my cpn for her to know the great work she does I would not be where I am just now if it was not for her lisa god bless you (https://www.careopinion.org.uk/331388)

Some pieces of feedback did contain mixed feedback, however, this often seemed to be used to emphasise or contrast against the person or organisation that the positive feedback referred to:

Would like to see more of my Care‐Coordinator (CCO), I feel they don't listen. Community psychiatric nurse (CPN) took time to listen to me. (https://www.careopinion.org.uk/1205919)

### 3.2 Intent

The majority of the feedback sought to express gratitude to individuals or teams. Herbland et al. (2017) state that expressions of gratitude are often in relation to the quality of care that has been experienced, whilst also recognising the emotional and professional labour of the work. People who had left feedback with the intention of gratitude also appeared to want to give something back to those who had cared for them and their families:

Ashley my sons mental health nurse is amazing if it weren't for the help of Ashley and his amazing team I now my son would not be here today and even though we still have a very long ongoing road ahead with my son, I will never be able to thank Ashley enough.

Thank you NHS for giving us nurses as amazing as Ashley and his mental health team (https://www.careopinion.org.uk/1275897)

Feedback also sought to emphasise change and highlight the impact of the work by individuals or teams on individual lives. The feedback highlighted perceptions of how different people felt their lives could have been without the contact with their mental health nurse:

She is this amazing person who made me feel comfortable. I'm not always comfortable around any medical professionals but she knew the person I am. To date, I still hate myself and I'm scared to face the world but the hate is a little less because of her (https://www.careopinion.org.uk/1086965)

Pieces of mixed feedback often sought to highlight the helpfulness of certain teams or individuals in comparison with others who were experienced as less helpful. This may be seen as a way in which individuals were attempting to mobilise their social capital (Day et al. 2020), as people receiving care, to catalyse change within health services:

I was seeing a staff member from STaRS but it wasn't helping me at all, I saw them 3 times in 4 months and it was making me drink more.

A different member of staff from stars then rang me and talked to me and actually got to know me and we worked out a plan going forward and now I feel I can beat this addiction.

I see a mental health nurse at park house. After I recently tried to take my own life she was brilliant with me, ringing me every day, then when I got out of hospital she brought my appointment forward and got me the help I needed (https://www.careopinion.org.uk/1254495)

### 3.3 Form

The narrative analysis revealed a typology comprised of four domains. These are: Narratives of being there, Narratives of listening and being heard, narratives of therapeutic relationships, Narratives of recovery.

**Table:** 

Dimension	Category	Category definition
Form—Narrative typology	Narratives of being there	Feedback relating to mental health nurses being present and supportive at a time of crisis or acute distress
	Narratives of listening and being heard	Feedback relating to the experience of feeling listened to and understood by mental health nurses
	Narratives of therapeutic relationships	Feedback relating to the quality of therapeutic relationships with mental health nurses
	Narratives of recovery	Feedback relating to the way in which mental health nurses had contributed towards recovery and change

#### 3.3.1 Narratives of Being There

These were narratives of mental health nurses being present and supportive during times of acute distress or crisis. These narratives often began with the author describing a time when their mental health had deteriorated significantly and they had needed to contact emergency services. The narratives provided an insight into the way in which mental health nurses were able to therapeutically respond to people in these moments:

I had a delirium, the mental health nurse was a great support to me +my wife. She listened to me and gave me good support at a time in my life that was a very scary experience. We both couldn't praise her enough. I just had a break down. I felt I was in a different world. I tried to harm myself (https://www.careopinion.org.uk/981007)

I was in a hospital outside of the area I live, which meant every week both my CPN [Community Psychiatric Nurse] and Psychologist travelled out of area to come and see me. Both came weekly religiously, communicated well with the ward staff and provided me with the best possible care. I'm almost certain that if it hadn't been for their support, I would have needed to stay in hospital longer than I did (https://www.careopinion.org.uk/55041)

These narratives also reflected the way in which mental health nurses worked with people throughout their recovery. Several of the narratives described the way in which the service user had been working with the mental health nurse on long term goals which had been achieved collaboratively since the period of crisis.

I had a major breakdown due to a number of issues and was struggling to cope. Adrian, my assigned psychiatric nurse visited me at home which was a massive help to me as at that point I was struggling to leave my house…. Adrian visited weekly and he listened to my concerns, gave advice and supported me without judging. He encouraged me to try different techniques to deal with my mental health issues and made me realise that giving up work and focusing on my health was probably the best way forwards. Thanks to my husband and Adrian I started taking baby steps by going out walking and I have progressed from there… (https://www.careopinion.org.uk/1225722)

Certain pieces of feedback related to the way in which mental health nurses were seen as not giving up on people and of offering consistent support despite the challenges that might arise during the journey to recovery:

The best ever care team!! Never gave up on me and stuck with me through everything. Always had the best CPN's, social workers and support staff!! Thank you (https://www.careopinion.org.uk/1136503)

For the past five years the Community Psychiatric Nurse from the Community Mental Health Team in North Ayrshire has been a tower of strength. He has steadfastly supported me in my recovery when I doubted my own survival. With his help I have gone onwards and upwards and have found my voice (https://www.careopinion.org.uk/463372)

#### 3.3.2 Narratives of Listening and Being Heard

These narratives described the way in which mental health nurses were able to foster a sense, with those they are working with, that they were genuinely listening to their concerns and supporting people to share their authentic emotions and responses:

She Is always helpful and considerate of my ever changing moods of depression, listening while I'm ranting then helping me make sense of negative thoughts of self‐harm e. g. overdosing, never dismissing these thoughts so I have no fear of expressing how I'm feeling to her because I know she won't deride me (https://www.careopinion.org.uk/284178)

The narratives often reflected an experience of being given time and an opportunity to explore emotions and concerns without a perceived time constraint. Some narratives described the mental health nurse in contrast to other professionals who were felt not to be listening:

He's extremely intelligent, very articulate and is seen by his mental health team as a great success. Yet no‐one wants to listen to his views except his wonderful community mental health nurse (https://www.careopinion.org.uk/991755)

Beyond the sense of being given time was also the frequently reported experience of feeling understood by mental health nurses. This seems to highlight a perception that both the service user and the mental health nurse had reached a shared understanding of symptoms, priorities, and service user wishes:

When we got to Carseview [hospital], Chloe and the nurse she was working with (I'm so sorry I can't remember her name), were patient, kind and empathetic, and I felt understood and cared for. Plans were put in place to ensure I was safe to go home that same night and I was able to go home knowing that help was there again if I needed it (https://www.careopinion.org.uk/1330177)

Feedback also captured the sense of compassion which people reported experiencing from mental health nurses:

Myself, my husband and my young family member, then had contact with the Hamilton Community Mental Health Team who were fantastic. The nurse allocated to us was amazing. They were kind, compassionate and navigated the difficult position with empathy and professionalism (https://www.careopinion.org.uk/1154900)

Helen was my Community Psychiatric Nurse for around 3 years. Looking back, that woman's patience is outstanding. I would get stuck in my ways a lot, going round in circles. I would be depressed and anxious and I never quite knew why. In the last couple of years it's begun to make sense. Helen's compassion and understanding made me feel safe. I had someone to trust and confide in while I was in a miserable situation (https://www.careopinion.org.uk/1167451)

#### 3.3.3 Narratives of Therapeutic Relationships

Many of the narratives described the relational nature of the work with mental health nurses, describing the strength and quality of the relationship. Several of the feedback narratives described a sense of anxiety or trepidation when being referred to mental health services but described the way in which mental health nurses quickly established a rapport with them:

Prior to commencing my online meetings I was very sceptical about attending this specialist type of treatment as I really felt I could cope on my own and that I would not benefit from someone telling me what or how to do things to make me feel and cope better. I felt it would be difficult for me to open up and speak to someone.

However, I have really enjoyed the sessions with my RMN who has been so kind, considerate and compassionate from day one. She made me feel at ease from day one and I feel I have built up a great rapport with her which led me to looking forward to and enjoying each session. It takes a special type of person to fulfill the role of those designated to do this job and my RMN certainly, without a doubt, is one of these genuine and caring nurses (https://www.careopinion.org.uk/970914).

I was a little apprehensive to start with, once the nurse arrived it all disappeared. The lady arrived, who was also a dog lover, and she showed me her own dog on her phone. She was absolutely lovely. It only took me a matter of a couple of minutes, and then I realised it wasn't an interrogation, it was a lengthy process though, the assessment was about 1.5 hours long. The mental health nurse really put me at ease—she has magic ways… (https://www.careopinion.org.uk/1090107).

Relationships were often described in terms of the mental health nurse going ‘above and beyond’, which often seemed to be related to the amount of time that people felt was given to them by the nurse and the impact this had upon the relationship:

My Community Psychiatric Nurse (CPN) comes once a week, every week; I often talking about difficult things to her and she is just so understanding and compassionate yet stays professional; always! I couldn't wish for better care than what I receive, always going above and beyond to help and support me no matter what! (https://www.careopinion.org.uk/1257450)

She has went above and beyond to ensure I'm safe and as happy as I can be. Our meetings are well structured and she is helping me to work through my issues and to get to the root of my troubles. She's kind, caring and compassionate to my situation and on top of our meetings she texts me almost every day to make sure I'm doing ok between our meetings (https://www.careopinion.org.uk/1275583)

The quality of the relationships described was often reported as experienced as safe, and not overtly clinical, but rather intrinsically human:

A different member of staff from STaRS then rang me and talked to me and actually got to know me and we worked out a plan going forward and now I feel I can beat this addiction (https://www.careopinion.org.uk/1254495)

They continue to support me giving me techniques to cope, a safe space to speak without any judgement and can also help to point out my thinking or perception. Or lack of at times (https://www.careopinion.org.uk/1225722)

#### 3.3.4 Narratives of Recovery

This theme related to the perceived progress that people had made in relation to their mental health. Feedback captured reflections on the impact that mental health nurses had made to their recovery:

I know I wouldn't still be here if this lady had not crossed my path. In the past on the occasion I have found some Mental Health support to be very abrupt and uncaring believe it or not. Supporting such very delicate people I have found this to be quite unbelievable, but being given Kim as my mental health support. I believe there must be a reason for me to remain walking this earth no matter how painful it can be…but with Kim by my side over these very traumatic 6 months have helped me go from a person living on emotional life support machine, to that same person in resting mode resting in a hospital bed…(this is my analogy of how I see myself from inside…at this time.) (https://www.careopinion.org.uk/1109447)

Some narratives spoke directly to the specific interventions which mental health nurses had been able to utilise to support recovery. These interventions ranged from medication to psychological interventions, and even very practical support with issues such as employment:

I had been on a medication for 4 years and the last six months I had been struggling again. I contacted my GP who looking at my medical history and referred me to my local mental health team. Kreshma was the name of the nurse, we talked through what was going on, there was no rush and she had time to make notes and talked about options for me. I was prescribed a different medication and have not felt so well in many years. (https://www.careopinion.org.uk/1264693)

My mental health nurse Lynette has truly been such an amazing support to me. Challenging my thinking, providing tools to make me think differently and to think more positively. She is so caring for people who she supports. I use the skills that she has taught me. To make my life so much more positive. I have progressed so much within her care of approximately 6 months because of what she has done for me. I am now able to volunteer at a mental health peer support organisation as with a view to paid employment (https://www.careopinion.org.uk/1262402)

I would like to express my gratitude to the Local Mental Health Team (LMHT) for their quick help and support when I recently lost my job. I contacted my Community Psychiatric Nurse (CPN) Dawn who within a few hours had compiled for me a letter of support for my appeal against losing my job. (https://www.careopinion.org.uk/1187373)

The narratives also spoke of looking forward to the future and the sense that mental health nurses had proactively contributed to their recovery.

Thank you for this service that is available to all who need it. When I started I felt my belly like a washing machine but with the help of Ann. I have reached down and switched the button off. I will move forward and be forever grateful to have gotten help from the service and Ann. Thank you. (https://www.careopinion.org.uk/1303181)

## 4 Discussion

There are those who argue that mental health nursing has become a profession which lacks focus, compassion, and therapeutic meaningfulness (McKeown 2024) and the positive feedback explored in this paper will not be reflective of all those engaging with mental health nurses. Only around 8% of people write online feedback, but when they do, this is often to give praise and highlight positive experiences (Brookes and Baker 2017; Powell et al. 2019). A study of 228 113 comments of online feedback posted on the NHS choices website in the UK revealed that words associated with a positive evaluation of experiences occurred three times more often than words associated with a negative evaluation. The results of this research found that people provided positive feedback as a way of expressing gratitude or emphasising the impact of mental health nurses on their lives. The typology of feedback suggests four domains consisting of Narratives of being there, Narratives of listening and being heard, Narratives of therapeutic relationships, and Narratives of recovery.

The sense of‘being there’ which was often highlighted in the data appears to relate to the way in which mental health nurses were often the professionals encountered during points of crisis. Joiner (2005) describes a sense of a lack of connectedness and belonging as an important dynamic in people attempting to end their lives. This sense of ‘being there’ perhaps speaks to the ability of mental health nurses to not only be present but to offer a sense of connection during the most distressing moments.

The feedback also highlighted the relational nature of the work of mental health nurses. The therapeutic relationship is acknowledged as an integral feature of psychological interventions and of value as a form of treatment in its own right (Curran et al. 2025). The relationship lays the foundation for interventions supporting people to meaningfully change their lives and address the multifaceted aspects of recovery. The feedback data also suggest the importance of longevity and continuity in the relationship and the way in which mental health nurses were able to sustain these relationships. This feedback further supports the wider evidence base on the importance of continuity of care (Weaver et al. 2017).

This research provides proof of concept evidence that knowledge can be gained about the impact of mental health nursing through the analysis of online feedback gathered through websites such as Care Opinion. It appears that people want to provide positive feedback about organisations and individuals (Powell et al. 2019), and websites such as Care Opinion provide an easy and accessible way for people to offer authentic feedback on their experiences with healthcare professionals. Whilst some concerns have been raised about online feedback in relation to content being potentially biased and generally negative (Patel et al. 2015), the findings indicate the potential value of the positive feedback and the insights it can provide into service user experience.

The environments where mental health nurses work are often highly emotive and carry a significant risk of moral distress and burnout (Musto and Schreiber 2012). Positive feedback can offer a meaningful reflection of the value of their work and an opportunity to experience a sense of professional pride and confidence (Stirling et al. 2023). However, there remains an absence of research exploring not only how to gather positive feedback but how to most effectively utilise this feedback to support staff in practice.

## 5 Conclusion

Positive feedback can provide a valuable insight into the experiences of service users accessing mental health services. This feedback is often provided with the intent to display gratitude or emphasise the positive change that has occurred as the result of individual or service involvement. The findings of this research emphasise the importance of the relational quality to mental health nursing, the importance of communication skills, the sense of ‘being there’ and the contribution to recovery. This research demonstrates the knowledge that can be gained from online feedback and suggests that further research is needed to better understand how the content of online feedback can be more robustly examined and integrated into mental health nursing policy and practice.

## 6 Relevance for Clinical Practice

Online feedback websites are becoming increasingly common and provide people with the opportunity to provide insights into their experiences of healthcare. Whilst there is a general assumption that the majority of the feedback will be negative, there is contradictory evidence to suggest that many people proactively leave positive feedback. This positive feedback can be valuable to mental health nurses, who continue to work in emotionally demanding environments and suffer frequent burnout. Further research is required to understand how positive feedback can most effectively support mental health nursing practice.

## Ethics Statement

Ethical approval was granted by the Faculty of Medicine and Health sciences research ethics committee at University of Nottingham, reference number: FMHS 291–0924.

## Conflicts of Interest

The authors declare no conflicts of interest.

## Data Availability

The data that support the findings of this study are available in Care Opinion at https://www.careopinion.org.uk/. These data were derived from the following resources available in the public domain: https://www.careopinion.org.uk/, https://www.careopinion.org.uk/.
